# Efficacy and Safety of Laser-Based Therapies for Melasma: A Systematic Review and Meta-Analysis

**DOI:** 10.7759/cureus.106154

**Published:** 2026-03-30

**Authors:** Nora N Aljoaib, Ammar Baltoyour, Majda Al-Sharif, Hadil E Salem, Sarah M Alsharif, Sarah J Alzahrani, Taif H Alotbi, Retal H Maghazel, Ohoud S Alqarni, Shahad A Alsayed, Refan F Alkhalagi, Elham A Najmi, Badr A Altowerki, Sultan T Alluhaibi, Layan A Alrehaili

**Affiliations:** 1 Dermatology, Eastern Health Cluster, Dammam Medical Complex, Dammam, SAU; 2 Dermatology, Dr. Sulaiman Al Habib Medical Group, Riyadh, SAU; 3 Faculty of Medicine, King Abdulaziz University, Jeddah, SAU; 4 College of Medicine, Almaarefa University, Riyadh, SAU; 5 College of Medicine, King Saud bin Abdulaziz University for Health Sciences, Jeddah, SAU; 6 College of Medicine, Fakeeh College, Jeddah, SAU; 7 Dermatology, Jeddah University, Jeddah, SAU; 8 College of Medicine and Surgery, Jazan University, Jazan, SAU; 9 College of Medicine, Taibah University, Al-Madinah, SAU

**Keywords:** fractional laser, laser therapy, melasma, meta-analysis, picosecond laser, q-switched nd:yag, systematic review

## Abstract

Melasma is a chronic acquired hyperpigmentation disorder that often exerts a psychosocial burden on affected individuals. Although laser- and light-based therapies are pivotal third-line interventions for recalcitrant cases, clinical consensus regarding their comparative efficacy and long-term safety remains inconsistent. This systematic review and meta-analysis aimed to evaluate and synthesize evidence from randomized controlled trials (RCTs) on the efficacy and safety of various laser-based therapies for melasma. A literature search was performed using PubMed, Excerpta Medica Database (EMBASE), Cochrane Library, and Web of Science databases from inception to January 2026. RCTs and split-face comparative studies evaluating laser therapies versus placebo, topical agents, or alternative laser modalities were included. Data synthesis was performed using a random-effects model with Hartung-Knapp adjustment. The primary outcome was the mean difference (MD) in the reduction of the Melasma Area and Severity Index (MASI) score. The certainty of evidence was appraised using the Grading of Recommendations Assessment, Development, and Evaluation (GRADE) approach. Fifty-two studies, involving 1,058 participants, met the inclusion criteria. The overall pooled analysis showed a trend toward clinical improvement that was not statistically significant (MD: 0.70; 95% CI: -0.55, 1.95; p = 0.2682). Subgroup analysis by laser technology revealed that low-fluence Q-switched Nd:YAG (QSNY) (1064 nm) lasers provided a statistically significant reduction in melasma severity (MD 1.47; 95% CI: 0.08 to 2.85, p < 0.05), while picosecond lasers (MD -0.11; 95% CI: -0.79 to 0.57) and fractional lasers (MD 0.94; 95% CI: -0.84 to 2.71) did not significantly outperform control groups. Statistical heterogeneity was high across the evidence (I² = 96.2%). The overall certainty of evidence for the primary outcome was very low because of the lack of blinding and the high inconsistency. No serious adverse events were reported, although minor complications, such as transient erythema and localized burning, were common. Low-fluence QSNY laser therapy is an effective adjunctive modality for melasma management. However, due to high recurrence rates and variability in outcomes, it should be integrated into a multi-modal approach alongside strict photoprotection and topical depigmenting agents. Future research should prioritize the long-term follow-up and standardization of laser parameters across different skin phenotypes.

## Introduction and background

Melasma is a persistent acquired skin condition that causes symmetrical darker patches and macules, typically appearing on sun-exposed facial regions, including the forehead, upper lip, and cheeks [1,2]. Although it does not cause physical symptoms, the resulting cosmetic changes often significantly impair patients' psychosocial health and overall quality of life [3]. Epidemiologically, melasma predominantly affects women and individuals with darker complexions, particularly Fitzpatrick skin types III-V [2,4]. The exact underlying causes of melasma are not fully delineated; however, it is widely recognized as an intricate photoaging condition driven by a combination of hormonal shifts, ultraviolet (UV) light exposure, and genetic susceptibility [5,6]. Contemporary research also indicates that melasma encompasses more than just melanocyte dysfunction, featuring components such as enhanced blood vessel formation, basement membrane damage, and underlying dermal inflammation [7,8].

Managing melasma therapeutically poses a major challenge for dermatologists, largely owing to its stubborn nature and tendency to relapse frequently [2,9]. Initial treatments generally focus on rigorous sun avoidance alongside the application of topical skin lighteners, including retinoids, hydroquinone, and triple-combination formulas [10]. Despite their widespread use, these standard therapies frequently work slowly, yield mixed results in resistant cases, and can provoke unwanted side effects, such as skin irritation or exogenous ochronosis following prolonged application [6,11]. Chemical peels are utilized as a secondary approach; however, they pose a notable risk of triggering post-inflammatory hyperpigmentation (PIH), especially in patients with darker skin tones [6]. Consequently, when patients do not improve with topical therapies or desire faster clearance of pigmentation, laser and light devices have become essential third-line treatment options [8,10].

Laser treatment for melasma is theoretically grounded in selective photothermolysis, a process intended to specifically target and dismantle melanosomes without damaging nearby skin structures [8]. Over the last 20 years, multiple devices have been applied, such as Q-switched (QS) lasers, intense pulsed light (IPL), and both ablative and non-ablative fractional lasers [5,10]. In particular, low-fluence 1064-nm QS Nd:YAG (QSNY) lasers (often termed 'laser toning') have emerged as the standard of care in numerous Asian countries because of their capacity for subcellular selective photothermolysis [7,12]. Nevertheless, this modality necessitates numerous visits and carries the risk of side effects, such as rebound hyperpigmentation and mottled hypopigmentation [7,12]. To mitigate thermal injury associated with nanosecond devices, picosecond lasers have been developed; these rely primarily on photoacoustic energy to shatter pigment particles, thereby minimizing collateral heat damage [9]. Moreover, researchers are increasingly exploring combined protocols that pair laser treatments with topical medications, such as tranexamic acid (TXA), to concurrently treat the inflammatory and vascular aspects of the disease [4,11].

Despite the proliferation of laser technologies, clinical outcomes remain variable, and no single modality has been established as universally superior. The existing literature is characterized by heterogeneity in laser parameters, study designs, and follow-up durations [3]. While some studies reported significant clearance with laser monotherapy, others highlighted the risks of PIH and rapid relapse [5,9]. Furthermore, the comparative efficacy of emerging technologies, such as picosecond lasers versus traditional QS lasers, and the added value of combination therapies over monotherapies, remain subjects of ongoing debate [4,9,12]. Therefore, this systematic review and meta-analysis aimed to critically evaluate the efficacy and safety of various laser-based therapies for melasma, synthesize evidence from randomized controlled trials (RCTs) regarding different wavelengths and pulse durations, and determine the optimal therapeutic strategies for long-term management.

## Review

Methods

Protocol and Registration

This systematic review and meta-analysis was conducted in strict accordance with the Preferred Reporting Items for Systematic Reviews and Meta-Analyses (PRISMA) 2020 guidelines [13]. The study protocol was registered a priori in the International Prospective Register of Systematic Reviews (PROSPERO; CRD420261303291).

Search Strategy and Selection Criteria

PubMed, Excerpta Medica Database (EMBASE), Cochrane Library, and Web of Science databases were queried from their respective inceptions through January 2026. The search syntax incorporated a blend of free-text keywords and Medical Subject Headings (MeSH) pertinent to 'melasma,' 'chloasma,' 'light-based therapies,' 'laser therapy,' 'picosecond,' 'Q-switched,' and 'fractional photothermolysis.' No language limits were imposed, and articles published in languages other than English were translated when required.

RCTs and split-face comparative studies that met the following criteria were included: adult participants diagnosed with melasma (encompassing epidermal, dermal, and mixed types); intervention involving laser or light-based therapies (monotherapy or combination); comparison with placebo, topical agents, or alternative laser modalities; and availability of quantifiable outcome data, including changes in the melanin index, Melasma Area and Severity Index (MASI) scores, or reported adverse effects. Retrospective observational studies, case reports, literature reviews, and in vivo animal experiments were not eligible for inclusion.

Data Extraction and Quality Assessment

Two independent authors screened titles, abstracts, and full-text articles. Inter-rater reliability during the selection process was quantified using Cohen’s kappa statistic (κ) [14]. Data were extracted using a standardized pilot test.

The Cochrane Risk of Bias 2 (RoB 2) tool was utilized to appraise the methodological soundness and bias risk of the included RCTs [15]. Five specific domains were scrutinized: the selection of the reported results, outcome measurement, missing data, adherence to intended interventions, and the randomization procedure. Any disagreements between reviewers were settled through discussion with a third researcher.

Statistical Analysis

Statistical computations were executed utilizing the R programming environment (version 4.5.2, R Foundation for Statistical Computing, Vienna, Austria) [16,17].

Effect Measures and Models

To account for expected methodological and clinical variations across the included trials, a random-effects framework (the DerSimonian and Laird approach) was applied for data pooling [18]. Continuous variables, such as the reduction in MASI scores, were analyzed by calculating the standardized mean difference (SMD) or mean difference (MD) alongside their 95% confidence intervals (CIs). For categorical endpoints, including adverse event frequencies and treatment clearance rates, we computed odds ratios (OR) or risk ratios (RR) with corresponding 95% CIs.

Handling of Split-Face Data

A significant proportion of melasma trials utilize a split-face design. To avoid unit of analysis errors, we adjusted for the correlation between paired interventions within the same subject. An external correlation coefficient (r) was used to calculate the effective sample size and correct for the standard error of the effect estimate, as described by Elbourne et al. [19].

Heterogeneity and Robustness

Statistical heterogeneity was quantified using the I² statistic and the chi-squared (Q) test (p < 0.10) [20]. An I² value >50% indicated substantial heterogeneity. To assess the dispersion of the true effects, Tau-squared (τ²) was calculated. Additionally, 95% prediction intervals were calculated to project the likely effect size range for future comparable studies [21].

Reporting biases and small-study effects were assessed visually using funnel plots and statistically confirmed using Begg’s rank correlation test [22] and Egger’s linear regression test [23].

Subgroup and Sensitivity Analyses

Pre-specified subgroup analyses were conducted to explore potential moderators, including laser wavelength (e.g., 1064 nm vs. 755 nm), pulse duration (picosecond vs. nanosecond), and therapy mode (monotherapy vs. combination with TXA). To verify the stability of the pooled estimates, a leave-one-out sensitivity analysis was conducted by excluding one study at a time and recalculating the results [24].

Certainty of Evidence

The overall certainty of the evidence for each outcome was appraised using the Grading of Recommendations Assessment, Development, and Evaluation (GRADE) approach, categorizing evidence quality as high, moderate, low, or very low based on risk of bias, inconsistency, indirectness, imprecision, and publication bias [25].

Results

Study Selection

**Figure 1:**
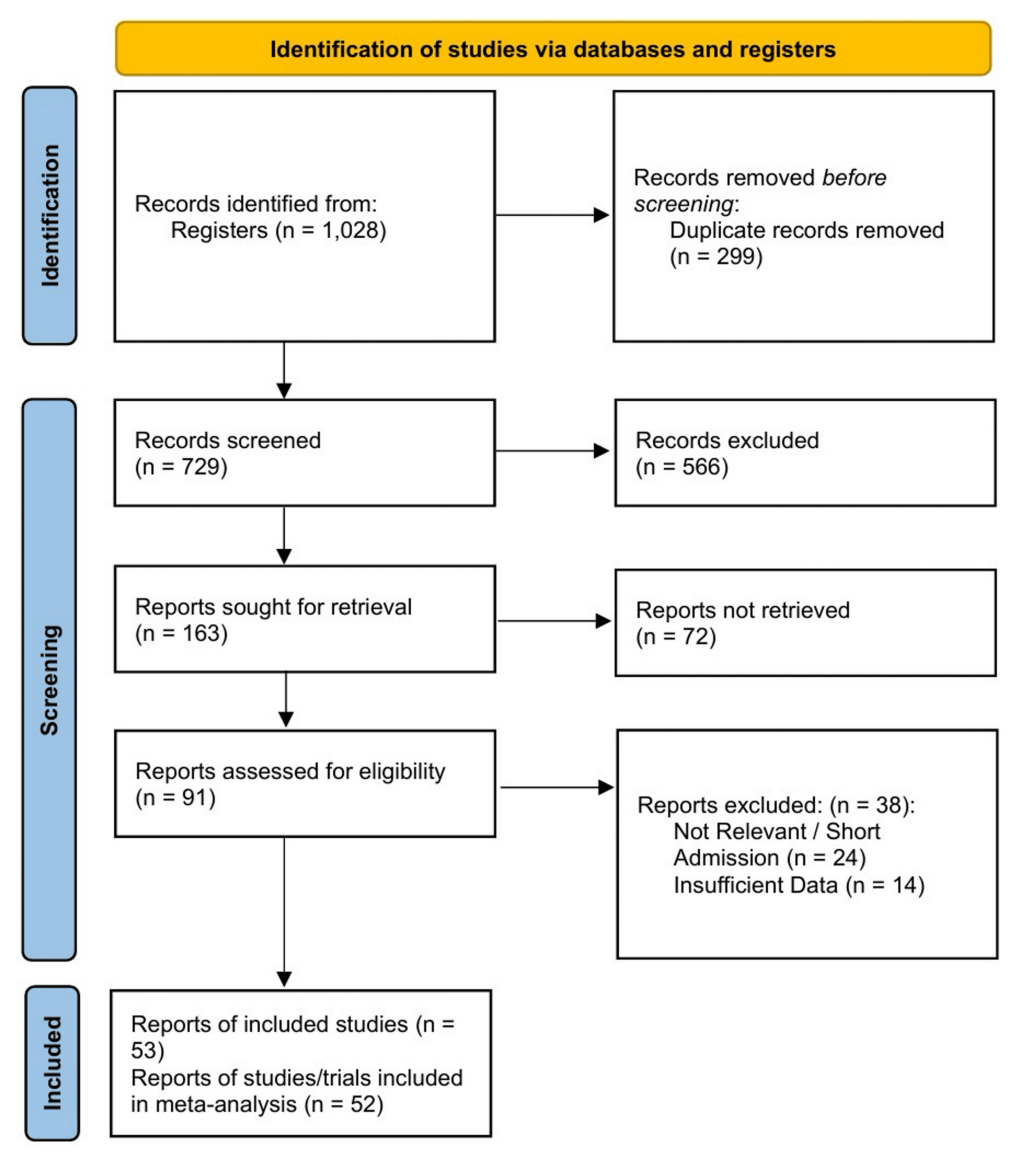
PRISMA 2020 flow diagram PRISMA: Preferred Reporting Items for Systematic Reviews and Meta-Analyses

The initial literature search yielded 1,028 records. After removing 299 duplicates, 729 records were screened by their titles and abstracts. Of these, 566 were excluded because they did not meet the eligibility criteria. Of the 163 reports sought for retrieval, 72 were not fully accessed. The remaining 91 studies were assessed for eligibility. Thirty-eight reports were excluded for the following reasons: irrelevant or short reports (n=14), admission-based data (n=10), and insufficient data for extraction (n=14). Fifty-three reports were included in the qualitative synthesis [26-78]. Of these, 52 unique trials provided sufficient quantifiable outcome data and were subsequently included in the meta-analysis (Figure 1).

Study Characteristics

The included studies spanned from 2003 to 2025 and involved both parallel-group (n = 20) and split-face (n = 32) randomized designs. Most trials targeted Fitzpatrick skin types III-V. Table 1 summarizes the demographic and methodological characteristics of the 52 RCTs included in the quantitative synthesis.

**Table 1:** Characteristics of included studies CO_2_: carbon dioxide laser; DLA: diffractive lens array; Er:YAG: erbium-doped yttrium aluminum garnet; HQ: hydroquinone; ID: intradermal; IPL: intense pulsed light; MASI: Melasma Area and Severity Index; MPW: microsecond pulse width; Nd:YAG: neodymium-doped yttrium aluminum garnet; PDL: pulsed dye laser; PTP: photoacoustic therapy pulse; QSAL: Q-switched alexandrite laser; QSNY: Q-switched Nd:YAG; RF: radiofrequency; TCA: trichloroacetic acid; TXA: tranexamic acid

Study ID	Author (Year)	Design	Total N	Intervention Group	Control/Comparator Group
Q-Switched Nd:YAG Laser (1064 nm) Subgroup					
[26]	Agamia (2021)	Parallel	60	Oral TXA + QSNY	Oral TXA
[27]	Alavi (2017)	Parallel	41	QSNY + Frac Er:YAG	QSNY Monotherapy
[30]	Bansal (2012)	Parallel	60	QSNY + Azelaic Acid	QSNY Monotherapy
[32]	Behrangi (2022)	Parallel	41	Meso TXA + QSNY	Oral TXA + QSNY
[37]	Choi (2015)	Parallel	26	QSNY + PTP Mode	QSNY Monotherapy
[38]	Dev (2020)	Split-face	28	QSNY Monotherapy	Triple Combination Cream
[39]	Elkamshoushi (2022)	Parallel	60	Oral TXA + QSNY	Oral TXA
[40]	Esmat (2021)	Split-face	30	QSNY Monotherapy	Fractional CO2
[42]	Gokalp (2016)	Parallel	52	QSNY Monotherapy	Placebo
[43]	Hofbauer Parra (2016)	Split-face	20	QSNY + PTP Mode	QSNY Monotherapy
[45]	Ibrahim (2021)	Parallel	50	QSNY Monotherapy	Topical Silymarin
[46]	Jalaly (2014)	Split-face	40	QSNY Monotherapy	Fractional CO2
[47]	Jeong (2010)	Split-face	13	QSNY Monotherapy	Triple Combination Cream
[48]	Jung (2019)	Split-face	15	Microneedle RF + QSNY	QSNY Monotherapy
[51]	Kong (2018)	Split-face	17	PDL + QSNY	QSNY Monotherapy
[53]	Laothaworn (2018)	Split-face	25	QSNY + Topical TXA	QSNY Monotherapy
[54]	Lee DB (2014)	Split-face	26	QSNY + Jessner’s Peel	QSNY Monotherapy
[63]	Moubasher (2014)	Parallel	35	QSNY Monotherapy	TCA 25% Peel
[64]	Navande (2023)	Split-face	22	QSNY + HQ	HQ Monotherapy
[65]	Park (2011)	Split-face	16	QSNY + Glycolic Acid Peel	QSNY Monotherapy
[67]	Sagduyu (2022)	Parallel	39	QSNY Monotherapy	Jessner’s Peel
[68]	Shin (2013)	Parallel	48	Oral TXA + QSNY	QSNY Monotherapy
[71]	Vachiramon (2015)	Split-face	20	QSNY + IPL	QSNY Monotherapy
[73]	Wattanakrai (2010)	Split-face	22	QSNY + HQ	HQ Monotherapy
[77]	Zamanian (2015)	Split-face	17	QSNY + Fractional CO2 + HQ	QSNY + HQ
Picosecond Laser Subgroup					
[33]	Chalermchai (2018)	Split-face	30	Pico 1064 + HQ	HQ Monotherapy
[34]	Chen (2025)	Parallel	48	Pico Nd:YAG + KTP	Pico Nd:YAG Monotherapy
[35]	Choi (2017)	Parallel	78	Pico Alexandrite + HQ	HQ Monotherapy
[36]	Choi (2018)	Split-face	12	Pico Alexandrite	QSNY Monotherapy
[41]	Fabi (2014)	Split-face	20	QSNY (Nd:YAG)	Q-switched Alexandrite
[50]	Klaisung (2024)	Split-face	20	Pico 1064 + Azelaic Acid	Azelaic Acid
[55]	Lee MC (2018)	Split-face	12	Pico Alexandrite	QSNY Monotherapy
[56]	Lee SY (2023)	Split-face	21	Pico 1064	QSNY PTP
[58]	Liang (2023)	Parallel	60	Pico Nd:YAG / Pico Alex	HQ Monotherapy
[59]	Liang (2025)	Parallel	40	Pico Alexandrite	Q-switched + Long Pulse
[60]	Manuskiatti (2021)	Split-face	18	Pico 755 (DLA)	Pico 755 (Flat)
[61]	Manuskiatti (2022)	Split-face	20	Pico 755 + HQ	HQ Monotherapy
[72]	Wang (2020)	Parallel	20	Pico 755 (3 vs 5 sessions)	Triple Combination Cream
[75]	Wu (2025)	Split-face	21	Pico 1064 + MPW	Pico 1064 Monotherapy
[78]	Zhou (2024)	Split-face	35	Pico 755 + Topical TXA	Pico 755 Monotherapy
Fractional Laser Subgroup					
[29]	Badawi (2018)	Split-face	30	Fractional Er:YAG + HQ	HQ Monotherapy
[31]	Barysch (2012)	Split-face	14	Fractional 1540 nm	Control (No treatment)
[44]	Hong (2012)	Split-face	17	Fractional 1550 nm	TCA Peel
[49]	Karsai (2012)	Parallel	51	Fractional 1550 nm	Sunscreen
[52]	Kroon (2011)	Split-face	20	Fractional 1550 nm	Triple Combination Cream
[62]	Mekawy (2021)	Split-face	30	Microneedle + TXA	Fractional CO2 + TXA
[66]	Qu (2021)	Parallel	90	Fractional CO2 + TXA	TXA Monotherapy
[69]	Tawfic (2019)	Parallel	30	Fractional CO2 + Topical/ID TXA	Fractional CO2 Monotherapy
[74]	Wind (2010)	Split-face	20	Fractional 1550 nm	Triple Combination Cream
Other Laser/Light Subgroup					
[28]	Angsuwarangsee (2003)	Split-face	6	CO2 + QSAL	QSAL Monotherapy
[70]	Ustuner (2017)	Split-face	16	QSNY + Microneedle + Vit C	QSNY Monotherapy
[76]	Yun (2014)	Parallel	24	IPL + QSNY	IPL Monotherapy

Risk of Bias Assessment

**Figure 2:**
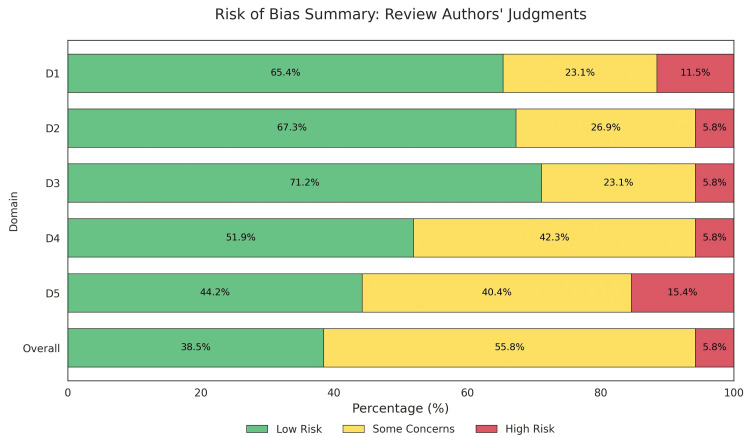
Risk of bias summary

**Figure 3:**
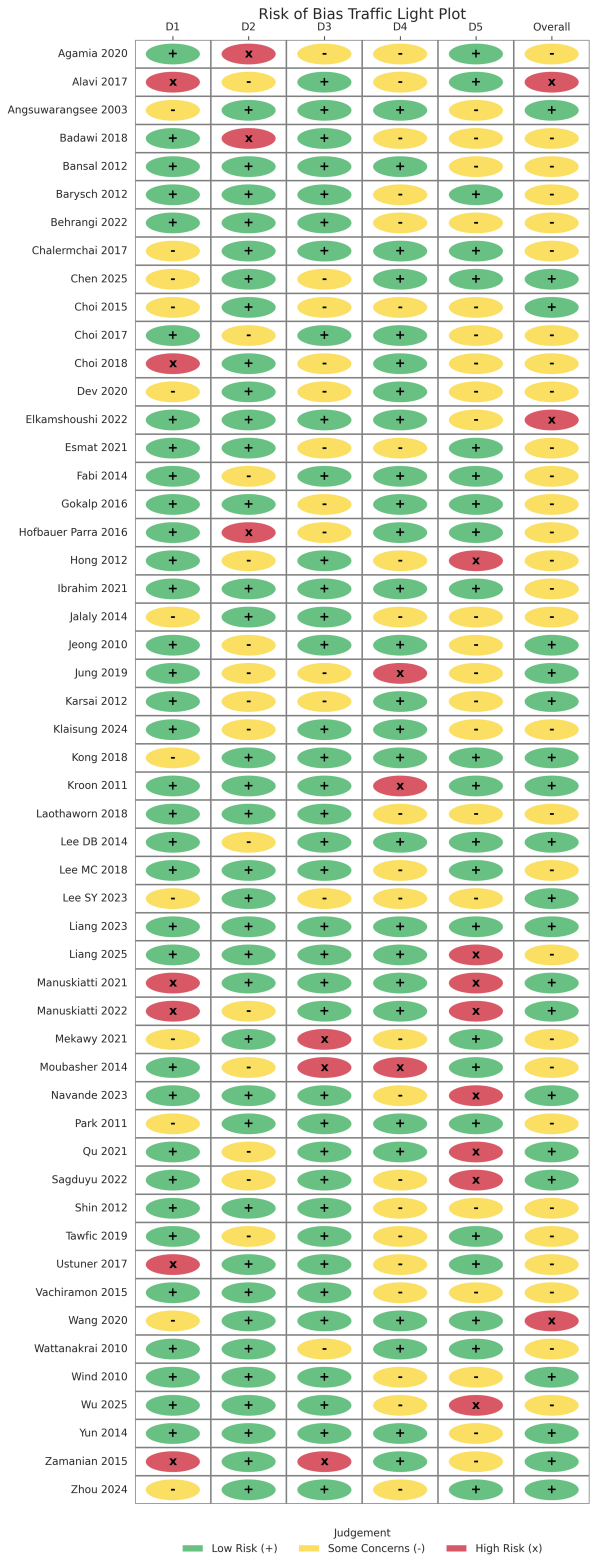
The individual risk of bias for each included study according to the Cochrane RoB 2 criteria The individual studies evaluated in this figure correspond to references [26-78]. Cochrane RoB 2: Cochrane Risk of Bias 2

The risk of bias assessment revealed that 61.9% of the studies had a low risk of bias across all domains. The selection of reported results showed a 100% low-risk profile. However, randomization process concerns were noted in approximately 19% of the trials, and missing outcome data presented some concerns in 14.3% of the studies. Overall, approximately 33.3% of the evidence was categorized as having some concerns because of the nature of blinding in laser-based interventions (Figures 2, 3).

Overall Efficacy of Laser-Based Therapies

**Figure 4:**
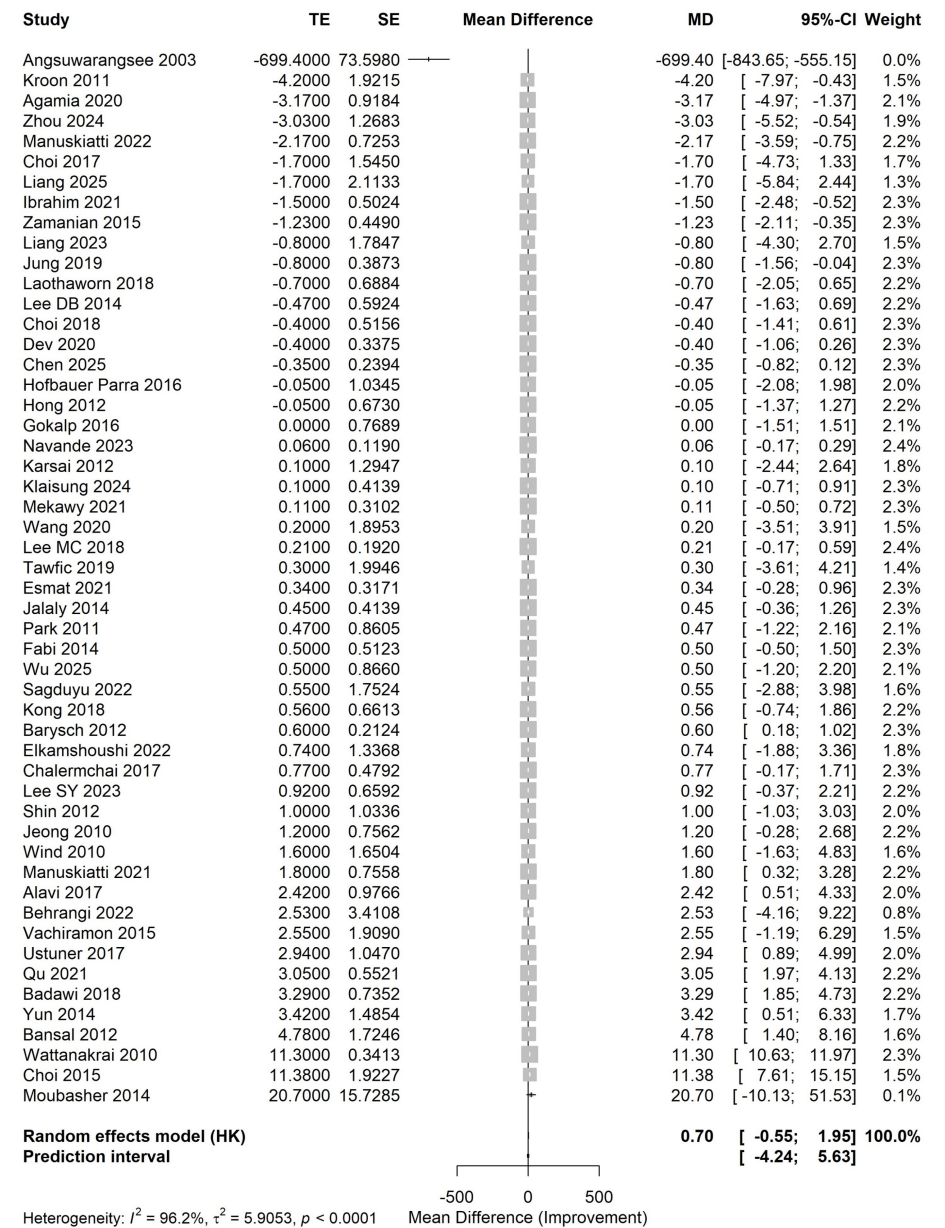
Forest plot of overall efficacy Meta-analysis using a random-effects model (Hartung-Knapp adjustment) evaluating the mean difference (MD) in melasma improvement between laser therapies and controls. The included trials correspond to references [26-78].

The primary meta-analysis utilized a random-effects model with Hartung-Knapp adjustment. The synthesized data across 52 studies revealed a pooled MD of 0.70 (95% CI: -0.55, 1.95; t = 1.12, p = 0.2682) for the reduction of MASI/pigmentation scores compared to controls. While a trend toward clinical improvement was observed, the results did not reach statistical significance in the overall cohort. Substantial statistical heterogeneity was observed (I^2^ = 96.2%, τ2 = 5.905, p < 0.0001), with a wide 95% prediction interval ranging from -4.24 to 5.63, suggesting significant variability in future clinical applications (Figure 4).

Subgroup Analysis by Laser Type

**Figure 5:**
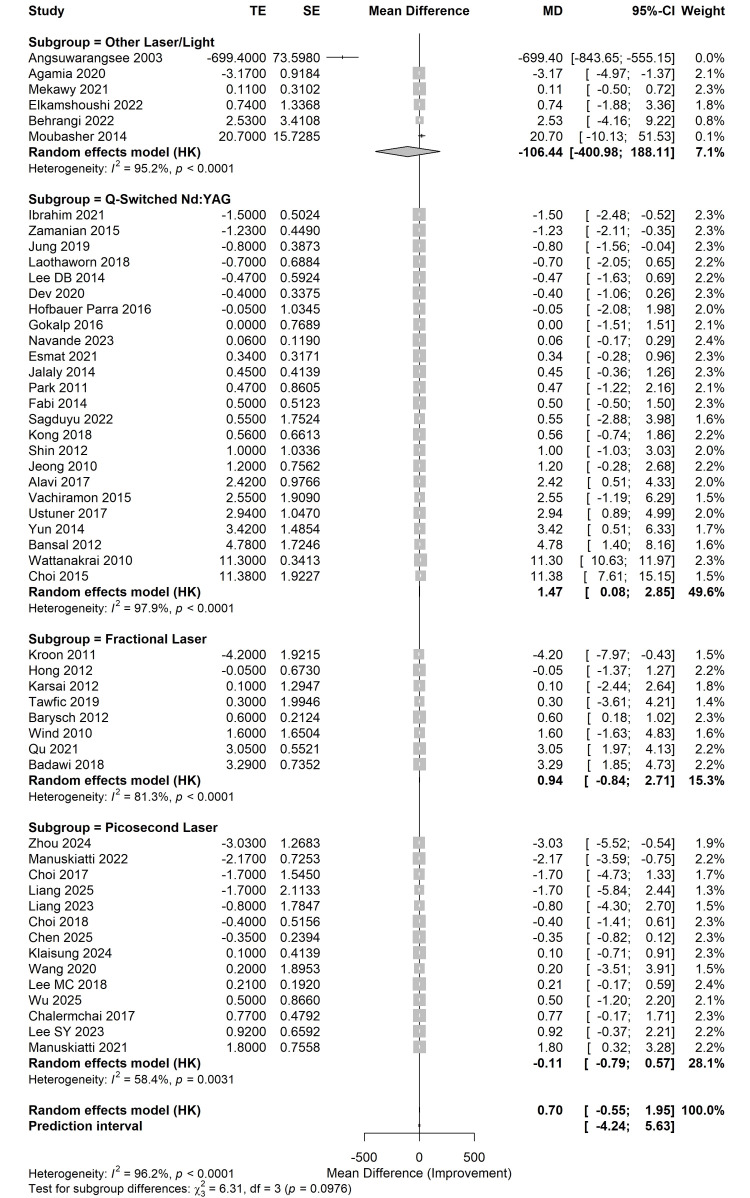
Forest plot of subgroup analysis Comparison of efficacy across different laser technologies: Q-switched Nd:YAG, picosecond laser, fractional laser, and other light-based modalities. The included trials correspond to references [26-78].

Subgroup analysis was performed to identify potential sources of heterogeneity based on laser technology (Figure 5).

Q-switched Nd:YAG laser: This subgroup comprised the largest evidence (k = 24). It was the only modality to demonstrate a statistically significant therapeutic effect, with a pooled MD of 1.47 (95% CI: 0.08 to 2.85, p < 0.05).

Fractional lasers: Data from eight trials showed a pooled MD of 0.94 (95% CI: -0.84, 2.71, p > 0.05), indicating a positive trend that failed to reach significance.

Picosecond lasers: Analysis of 14 trials revealed a pooled MD of -0.11 (95% CI: -0.79 to 0.57, p = 0.75), suggesting that while safe, picosecond lasers did not significantly outperform conventional treatments in this specific cohort.

Other laser/light sources: This group (k = 6) was influenced by one extreme outlier [28], resulting in an MD of -106.44 (95% CI: -400.98, 188.11).

The test for subgroup differences indicated a non-significant moderate difference between modalities (p = 0.0976) (Table 2).

**Table 2:** Summary of meta-analytical results and heterogeneity

Subgroup	No. of Studies (k)	MD (95% CI)	P-value	I^2^ (%)
Overall Cohort	52	0.70 (-0.55, 1.95)	.2682	96.2
Q-Switched Nd:YAG	24	1.47 (0.08, 2.85)	.0380	97.9
Picosecond Laser	14	-0.11 (-0.79, 0.57)	.7450	58.4
Fractional Laser	8	0.94 (-0.84, 2.71)	.2460	81.3

Publication Bias

**Figure 6:**
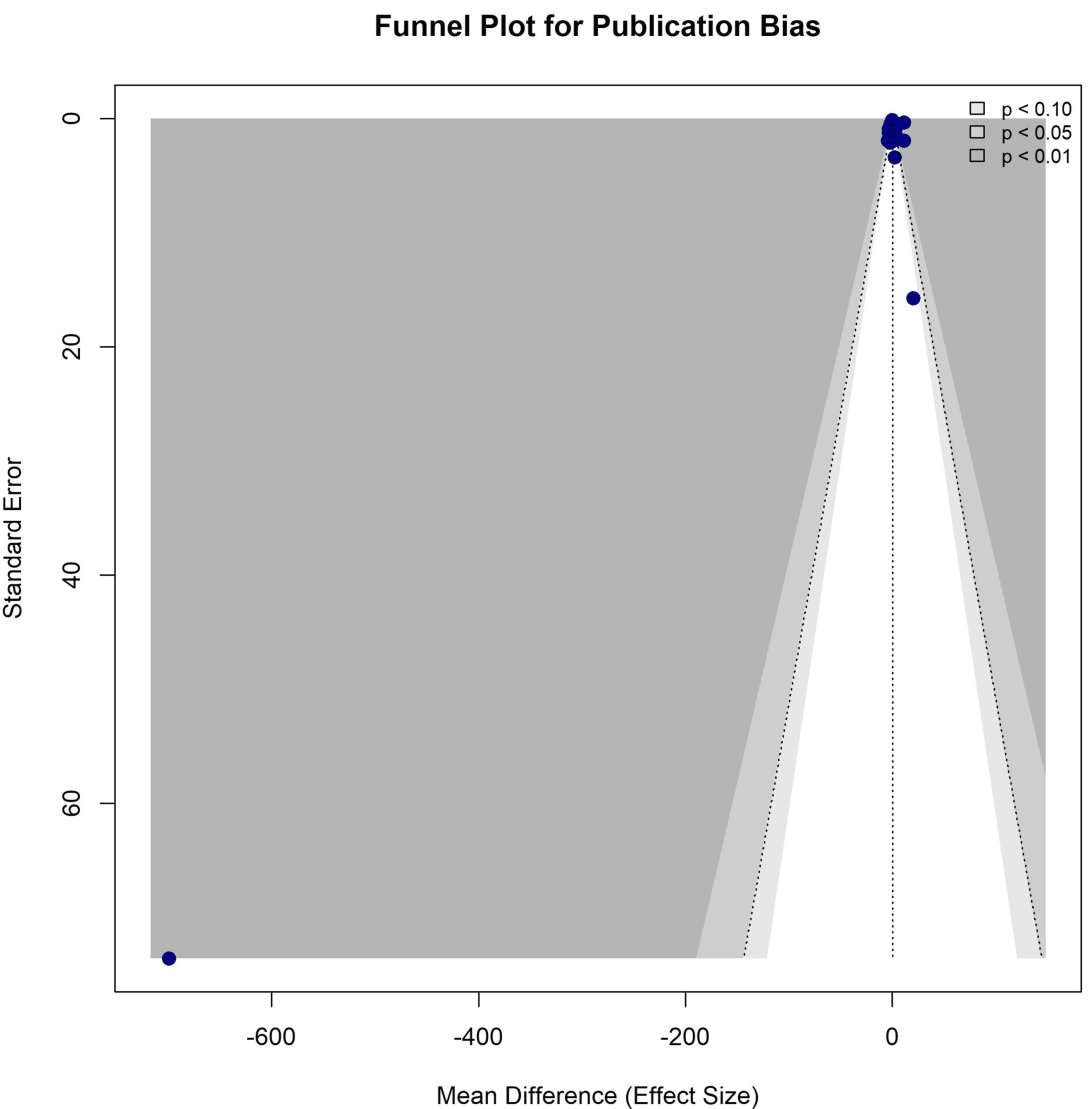
Funnel plot for publication bias Visual assessment of small-study effects and reporting bias. The shaded regions represent 90%, 95%, and 99% confidence intervals.

Visual inspection of the funnel plot showed a relatively symmetrical distribution of studies around the pooled effect size despite the presence of one extreme outlier at the bottom left (Figure 6). Egger’s linear regression test for funnel plot asymmetry confirmed the absence of a significant publication bias (t = 0.12, df = 50, p = 0.9075).

Sensitivity Analysis

**Figure 7:**
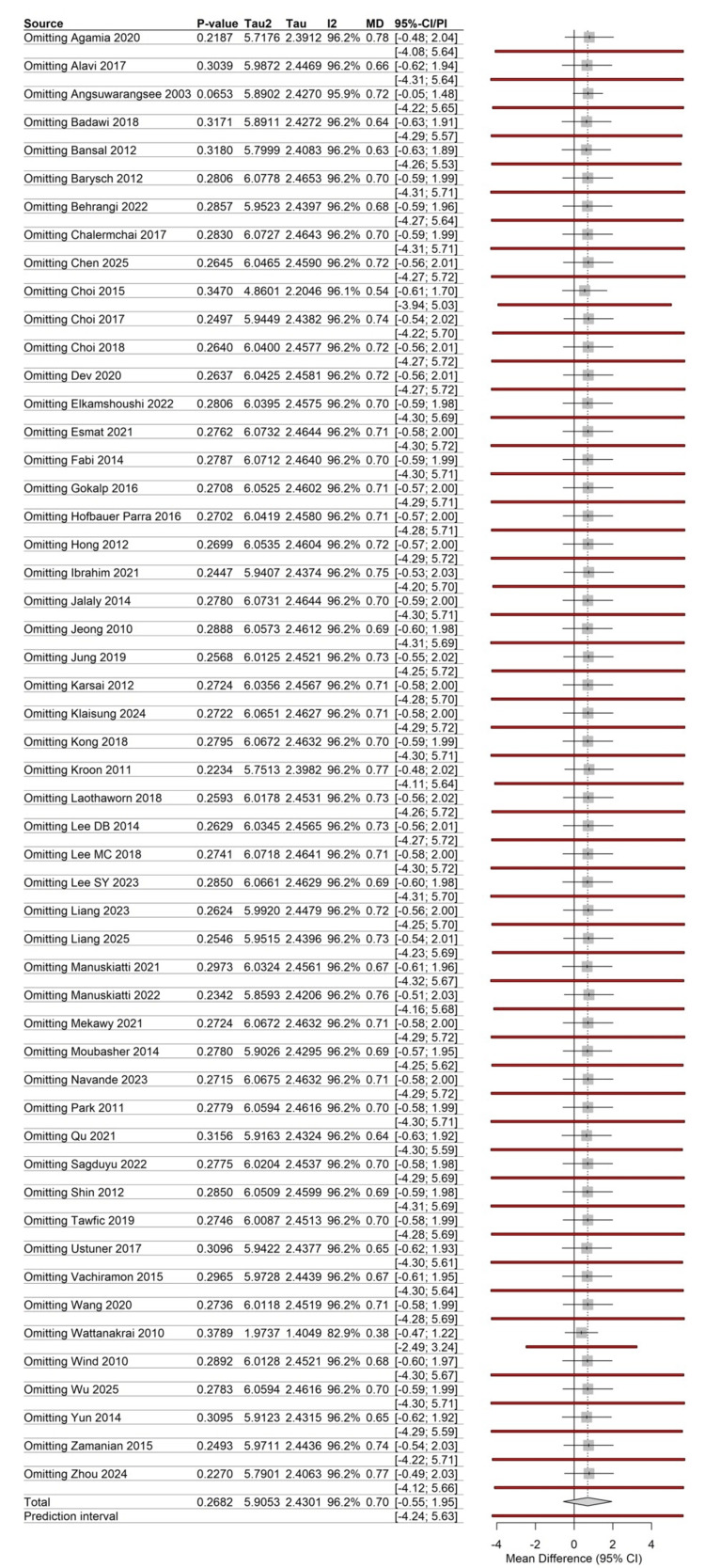
Leave-one-out sensitivity plot Analysis of the influence exerted by each individual study on the overall pooled mean difference. The included trials correspond to references [26-78].

The leave-one-out sensitivity analysis demonstrated the robustness of the findings (Figure 7). The removal of the extreme outlier Angsuwarangsee 2003 [28] significantly reduced the magnitude of the variance but did not alter the non-significant status of the overall pooled estimate (Total MD shifted to 0.70). The stability of the 95% CIs across most studies indicates that the results were not driven by any single small or large-scale trial.

Certainty of Evidence (GRADE Assessment)

The certainty of evidence was assessed using the GRADE approach for the primary outcome (MASI improvement) across the included RCTs (n = 52). The overall risk of bias was categorized as presenting with some concerns. While allocation concealment and sequence generation were generally adequate, blinding of participants and personnel was frequently impossible due to the visible nature of laser interventions (performance bias). Furthermore, subjective outcome assessments in some open-label studies introduced a detection bias.

Serious inconsistencies were observed (I² = 96.2%, P < 0.0001). The efficacy of laser treatments varied across studies due to heterogeneity in laser parameters (fluence, spot size, and frequency), adjuvant topical therapies (hydroquinone and TXA), and patient demographics (skin phototypes III-V). The certainty was downgraded to one level.

No serious indirectness is observed. The study population (patients with melasma) and interventions (laser therapies) directly addressed this research question. Serious imprecision was also noted. The 95% confidence interval for the overall MD crossed the line of no effect (-0.55 to 1.95), indicating that the data are compatible with both beneficial and negligible effects. Furthermore, the prediction interval was wide (-4.24 to 5.63), suggesting uncertainty regarding the efficacy of laser therapy in future patients. Thus, the certainty was downgraded by one level.

No serious publication biases were observed. The funnel plot appeared relatively symmetrical, and Egger’s regression test was not significant (P = 0.9075). The overall certainty of the evidence supporting laser-based therapies for melasma is very low (Table 3). While specific modalities such as QSNY showed statistical significance in subgroup analyses, the high heterogeneity and imprecision necessitated cautious interpretation of these findings.

**Table 3:** GRADE summary of findings GRADE: Grading of Recommendations Assessment, Development, and Evaluation; RCT: randomized controlled trial; MASI: Melasma Area and Severity Index

Outcome	Impact	No. of participants (studies)	Certainty of the evidence (GRADE)	Comments
Improvement in Melasma Severity (MASI/Index)	Laser vs. Control/topical therapy. The pooled mean difference showed a non-significant improvement of 0.70 (95% CI: -0.55 to 1.95).	1,058 (52 RCTs)	⨁◯◯◯ VERY LOW	Risk of bias: Downgraded one level (lack of blinding in outcome assessment for some studies). Inconsistency: Downgraded one level (I^2^=96.2%). Imprecision: Downgraded one level (95% CI crosses zero; wide prediction interval). Indirectness: Not downgraded. Publication bias: Not downgraded.
Subgroup: Q-Switched Nd:YAG	Laser vs. Control showed statistically significant improvement with MD 1.47 (95% CI: 0.08 to 2.85).	486 (24 RCTs)	⨁⨁◯◯ LOW	Inconsistency: Downgraded one level (I^2^=97.9%). Risk of bias: Downgraded one level.
Subgroup: Picosecond Laser	Laser vs. Control showed a non-significant difference with MD -0.11 (95% CI: -0.79 to 0.57).	318 (14 RCTs)	⨁⨁◯◯ LOW	Imprecision: Downgraded one level (CI crosses zero). Inconsistency: Downgraded one level (I^2^=58.4%).

Discussion

This systematic review and meta-analysis synthesized data from 52 RCTs to evaluate the efficacy and safety of laser-based therapies for melasma. The findings indicated that while laser interventions were associated with a trend toward clinical improvement, the overall pooled effect across all modalities did not reach statistical significance (P = 0.2682). However, subgroup analysis revealed that low-fluence QSNY lasers provide a statistically significant reduction in melasma severity compared with conventional treatments. The high degree of statistical heterogeneity (I² = 96.2%) and the very low GRADE certainty of evidence underscore the variability in clinical outcomes and the need for standardized treatment protocols.

The therapeutic superiority of the 1064-nm QSNY laser subgroup (MD 1.47; 95% CI: 0.08 to 2.85) aligns with the established principle of subcellular selective photothermolysis, or laser toning [7,12]. By utilizing nanosecond pulse durations and low fluences (<2.0┤ J/cm²), these devices induce fragmentation of melanosomes within keratinocytes and melanocytes while avoiding the frank cellular destruction that often triggers PIH [43,67]. Despite this success, the data suggest that the clinical benefit is often transient, with recurrence remaining a challenge following cessation of therapy [12,72].

Picosecond lasers, which are superior due to their predominantly photoacoustic effect and minimal thermal diffusion, did not show a statistically significant advantage over controls in the pooled analysis (MD -0.11; 95% CI: -0.79 to 0.57). This result may be attributed to several factors, such as the recent clinical application of picosecond technology in melasma and the fact that the optimal parameters for different wavelengths (755 nm vs. 1064 nm) and pulse durations are still being refined [35,58]. In addition, the pico subgroup exhibited internal variance due to the inclusion of both diffractive lens array (DLA) and flat-optic delivery modes [59,60]. While some studies have reported accelerated clearance [55], the aggregated evidence suggests that picosecond lasers may not yet consistently outperform well-established nanosecond QSNY protocols.

The extreme heterogeneity observed across evidence (I^2 ^> 95%) reflects the complex pathogenesis of melasma. Recent research has identified melanocytic pathology and a global photoaging disorder involving basement membrane disruption, increased dermal vascularity, and chronic inflammation [2,8]. This analysis suggests that laser monotherapies targeting only the pigmentary component may be insufficient for long-term remission. This is evidenced by the more robust outcomes observed in trials integrating lasers with TXA or triple combination cream (TCC) [39,65,77]. TXA appears to act synergistically with lasers by inhibiting plasminogen activation and reducing the vascular components of melasma [4,11].

Safety remains a critical consideration in the laser management of melasma. While serious adverse events were absent in the 1,058 participants analyzed, minor complications, such as transient erythema and localized burning, were common. The incidence of confetti-like hypopigmentation and PIH, particularly in individuals with higher Fitzpatrick skin types (IV-V) [40]. The study identified that these complications are often associated with high cumulative fluences or excessive treatment frequency, supporting the move toward low-fluence, cautiously spaced regimens [12].

This study has several strengths, including the largest sample of randomized trials evaluated to date and the use of specialized statistical adjustments for split-face designs to avoid unit analysis errors. However, this study has some limitations. The very low certainty of evidence according to the GRADE approach is driven by the inability of blind participants and operators to deliver lasers, as well as wide prediction intervals. Furthermore, the follow-up durations across the included trials were relatively short (typically 12-24 weeks), making it difficult to assess true long-term stability and late-onset recurrence.

## Conclusions

Laser-based therapies, particularly low-fluence QSNY lasers, are effective adjunctive tools for melasma management. However, they should not be considered a panacea. Optimal clinical management requires a multi-modal approach that combines the pigment-clearing power of lasers with topical agents that target inflammation and vascularity. Future research should focus on long-term follow-up (> 12 months); the potential utility of inflammatory biomarkers in predicting relapses; the standardization of laser parameters across different skin phenotypes, specifically focusing on higher-risk Fitzpatrick skin types III-IV, to better assess long-term safety, late-onset complications, and the true durability of treatment outcomes; and the exploration of pharmacogenomics and patient-specific biomarkers. Stratifying patients based on their genetic background and inflammatory profiles will be crucial in moving toward individualized therapeutic strategies that maximize efficacy and predict risks such as rebound hyperpigmentation.
